# Enhanced Vascular Contractility Following Secondhand Smoke Exposure: A Pathological “Double-hit” to Critical Smooth Muscle Ion Channels

**DOI:** 10.1093/function/zqab061

**Published:** 2021-11-11

**Authors:** Nicholas R Klug, Mark T Nelson

**Affiliations:** Department of Pharmacology, University of Vermont, 05405 Burlington, VT, USA; Department of Pharmacology, University of Vermont, 05405 Burlington, VT, USA; Division of Cardiovascular Sciences, University of Manchester, M139WU Manchester, UK

## A Perspective on “Secondhand Smoke Exposure Impairs Ion Channel Function and Contractility of Mesenteric Arteries”

Decades of mechanistic, translational, and clinical effort have been spent attempting to understand the link between smoking and vascular function. Indeed, these studies have clearly shown that smoking is a cause of many cardiovascular diseases, such as atherosclerosis, hypertension, stroke, and peripheral arterial disease.^1,2^ However, much less attention has been paid to the vascular effects of *secondhand* smoke exposure, and the studies that have examined this issue have primarily focused on endothelial dysfunction. Endothelial dysfunction is certainly a crucial locus of vascular disease. However, a mechanistic examination of smooth muscle cells—the other major vascular cell type—could deepen our extremely shallow understanding of secondhand smoke-related vascular dysfunction.

In this issue's article, “Secondhand Smoke Exposure Impairs Ion Channel Function and Contractility of Mesenteric Arteries,”^3^ Le *et al*. provide elegant inroads into this question. The authors of this article demonstrate that chronic exposure of mice to secondhand smoke for 12 weeks caused an increase in the myogenic tone of mesenteric arteries. To tackle the mechanisms underlying this intrinsic elevation in contractility, they first utilized patch-clamp electrophysiology on isolated arterial smooth muscle cells, revealing a clear increase in L-type calcium channel (Ca_V_1.2) activity at single-channel resolution. They go on to show that this likely occurs through increased calcium channel expression and clustering.^4^ Le and colleagues then nicely demonstrate that BK_Ca_ channel activity is decreased in smooth muscle cells, likely through regulation by the calcium-dependent transcription factor NFAT (nuclear factor of activated T cells). Critically, this loss of basal potassium efflux through BK_Ca_ channels resulted in a more depolarized resting membrane potential. In short, the authors conclude that secondhand smoke exposure increases arterial contractility by amplifying voltage-dependent calcium influx in smooth muscle cells.

From a vascular function standpoint, this creates an alarming “double-hit” scenario: suppression of BK_Ca_ channel activity leaves smooth muscle cells in a relatively depolarized, “primed” state; this depolarization then further enhances intrinsic increases in L-type calcium channel conductivity.

Putting these specific results in perspective, the two channels that are potently altered by secondhand smoke exposure are intimately linked and serve as key players in basic vascular smooth muscle function. The smooth muscle BK_Ca_ channel is a crucial element for establishing resting membrane potential and can also dynamically regulate membrane potential in response to many stimuli,^5^ whereas activity of the smooth muscle L-type calcium channel, which is linked to BK_Ca_ channel function through its voltage dependence, is the overwhelming source of calcium for calcium-mediated contractility.^6^ The linkage of these two channels also manifests in the pathology under investigation in that Ca_V_1.2-mediated calcium entry enhances NFAT-mediated downregulation of BK_Ca_ channel expression/function. Directly examining the contribution of the well-known calcium/calcineurin-NFAT axis to this linkage and determining whether other mechanisms apart from calcineurin-mediated NFAT activation regulate BK_Ca_ channel and/or calcium channel function are worthy avenues of future study.

This report by Le *et al*. provides a carefully elucidated, biophysical basis for vascular dysfunction following secondhand smoke exposure. But equally important, they provide a foundation for addressing many important unanswered questions. Some of these are relatively specific—What effect do these changes have on whole animal vascular function, such as blood pressure regulation and activity-dependent increases in blood flow (ie, functional hyperemia)? What are the effects of these changes on the autonomic nervous system^7^ or other vascular beds? Are there other channels in play, either through their direct influence on membrane voltage/intracellular calcium or by enhancing channel clustering properties?^8^ And what are the specific particulates or compounds in secondhand smoke that drive this pathophysiological response? Other questions are broader—Is vascular ion channel function also altered during exposure to more persistent environmental air pollutants?^9^ And how does manifestation of the pathology depend on the timing of introduction to secondhand smoke (eg, childhood versus adulthood)? Overall, this article provides the type of mechanistic characterization that is critical for ultimately preventing or treating vascular disease and will serve as a foundation for future efforts in the field to tackle the complex origins of vascular dysfunction.
